# MIXed plastics biodegradation and UPcycling using microbial communities: EU Horizon 2020 project MIX-UP started January 2020

**DOI:** 10.1186/s12302-021-00536-5

**Published:** 2021-08-21

**Authors:** Hendrik Ballerstedt, Till Tiso, Nick Wierckx, Ren Wei, Luc Averous, Uwe Bornscheuer, Kevin O’Connor, Tilman Floehr, Andreas Jupke, Jürgen Klankermayer, Luo Liu, Victor de Lorenzo, Tanja Narancic, Juan Nogales, Rémi Perrin, Eric Pollet, Auxiliadora Prieto, William Casey, Thomas Haarmann, Alexandru Sarbu, Ulrich Schwaneberg, Fengxue Xin, Weiliang Dong, Jiamin Xing, Guo-Qiang Chen, Tianwei Tan, Min Jiang, Lars M. Blank

**Affiliations:** 1 Institute of Applied Microbiology (iAMB), Aachen Biology and Biotechnology (ABBt), RWTH Aachen University, Worringerweg 1, 52074 Aachen, Germany; 2 Institute of Biotechnology (BIOTEC), Aachen Biology and Biotechnology (ABBt), RWTH Aachen University, Worringerweg 3, 52074 Aachen, Germany; 3 Institute of Technical and Macromolecular Chemistry (ITMC), RWTH Aachen University, Worringerweg 2, 52074 Aachen, Germany; 4 Fluid Process Engineering, Aachen Process Technology (AVT), RWTH Aachen University, Forckenbeckstraße 51, 52074 Aachen, Germany; 5 Institute of Bio- and Geosciences IBG-1: Biotechnology, Research Center Jülich, Wilhelm Johnen Straße, 52428 Jülich, Germany; 6 Institute of Biochemistry, University of Greifswald, Felix-Hausdorff-Str. 4, 17487 Greifswald, Germany; 7 BiOrbic Bioeconomy SFI Research Centre, UCD Earth Institute and School of Biomolecular and Biomedical Science, University College Dublin, Belfield, Dublin 4, Ireland; 8 Interdisciplinary Platform for Sustainable Plastics Towards a Circular Economy-Spanish National Research Council (SusPlast-CSIC), Biological Research Center (CIB-CSIC), 28040 Madrid, Spain; 9 BioTeam/ICPEES-ECPM, UMR CNRS 7515, Université de Strasbourg, 25 rue Becquerel, 67087 Strasbourg Cedex 2, France; 10 SOPREMA, Direction R&D, 14 Rue Saint Nazaire, 67100 Strasbourg, France; 11 Bioplastech Ltd., Nova UCD, Belfield Innovation Park, University College Dublin, Belfield, Dublin 4, Ireland; 12 AB Enzymes GmbH, Feldbergstraße 78, 64293 Darmstadt, Germany; 13 everwave GmbH, Strüverweg 116, 52070 Aachen, Germany; 14 College of Biotechnology and Pharmaceutical Engineering, Nanjing Tech University, No. 30 Puzhu Road, Nanjing, 211816 PR China; 15 School of Life Sciences (SLS), Tsinghua University, Beijing, 100084 PR China; 16 State Key Laboratory of Biochemical Engineering, Institute of Process Engineering (IPE), Chinese Academy of Sciences, 1 North 2nd Street, Zhongguancun, Beijing, 100190 PR China; 17 College of Life Science and Technology (CLST), Beijing University of Chemical Technology, Beisanhuan EastRoad 15, Chaoyang District, Beijing, 100029 PR China

**Keywords:** PHA, Polyhydroxyalkanoate, Synthetic biology, Plastic recycling, Plastic crisis, Metabolic engineering, Microbial consortia, Mixed plastics valorisation, Biobased plastic, Depolymerisation

## Abstract

This article introduces the EU Horizon 2020 research project MIX-UP, "Mixed plastics biodegradation and upcycling using microbial communities". The project focuses on changing the traditional linear value chain of plastics to a sustainable, biodegradable based one. Plastic mixtures contain five of the top six fossil-based recalcitrant plastics [polyethylene (PE), polyurethane (PUR), polypropylene (PP), polyethylene terephthalate (PET), polystyrene (PS)], along with upcoming bioplastics polyhydroxyalkanoate (PHA) and polylactate (PLA) will be used as feedstock for microbial transformations. Consecutive controlled enzymatic and microbial degradation of mechanically pre-treated plastics wastes combined with subsequent microbial conversion to polymers and value-added chemicals by mixed cultures. Known plastic-degrading enzymes will be optimised by integrated protein engineering to achieve high specific binding capacities, stability, and catalytic efficacy towards a broad spectrum of plastic polymers under high salt and temperature conditions. Another focus lies in the search and isolation of novel enzymes active on recalcitrant polymers. MIX-UP will formulate enzyme cocktails tailored to specific waste streams and strives to enhance enzyme production significantly. In vivo and in vitro application of these cocktails enable stable, self-sustaining microbiomes to convert the released plastic monomers selectively into value-added products, key building blocks, and biomass. Any remaining material recalcitrant to the enzymatic activities will be recirculated into the process by physicochemical treatment. The Chinese–European MIX-UP consortium is multidisciplinary and industry-participating to address the market need for novel sustainable routes to valorise plastic waste streams. The project's new workflow realises a circular (bio)plastic economy and adds value to present poorly recycled plastic wastes where mechanical and chemical plastic recycling show limits.

## Background

### General global plastic waste situation

**Fig. 1:**
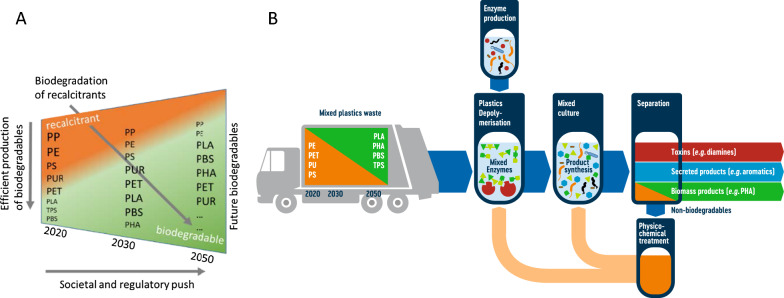
**A** The MIX-UP ambition to make the majority of the vast plastics biodegradable. **B** Overall schematic concept of the MIX-UP-project: mechanically pre-treated, unsorted plastic waste is exposed during *Plastics Depolymerisation* to heavily engineered, plastic type-specific enzyme blends. These enzymes have been produced preliminarily by defined mixed cultures during *Enzyme production*. Released metabolites, additives, plastic mono- and oligomers of various plastic types will be used as plastic derived feedstock for defined microbial communities (*Mixed culture*) to bio-convert them into biomass products (e.g. PHA), and secreted products released during upcycling processes, respectively. During *Separation,* downstream processing, removal of toxin (e.g. diamines) and product recovery will be tackled. Finally, recalcitrant residues will be subjected to *Physicochemical treatments*, thereby synthesising added-value products and closing MIX-UP cycle by re-entering into bioprocess steps

Due to their benefits as a functional material, their extreme durability, longevity, low weight and low price, synthetic plastics, including polyvinyl chloride (PVC), PE, PS, PP, PUR, and PET, have become ubiquitous not only in work and social environments, but also in natural systems as contaminants. Plastic pollution has become a global threat, affecting all ecosystems, even remote ones like the pole regions, uninhabited atolls or deep ocean basins [1–6]. The global scale of plastics production increased by 21% in the last six years, reaching 368 million metric tons (Mt) in 2019 [7]. China and the European Union (EU) account for 31% and 16%, ranking first and third globally of all the world's plastic production, respectively. Nonetheless, European plastic production revealed a decline since 2018 which has been significantly intensified by COVID-19 pandemic (estimated rate 2020: − 8.5%) [7]. Highest in plastic waste generation in 2016 were the United States, with 42 Mt followed by the EU (30 Mt), India (26 Mt) and China (22 Mt) [8]. All nations worldwide are struggling to manage the current volumes of plastic waste, making a highly efficient waste management system increasingly important. A significant unintended drawback of the existing plastic economy is its linearity. Of all plastics produced globally, 83% has not been reused due to a lack of proper recycling technologies, and of the recycled 10%, only 15% has been reused more than once [9]. Seven main plastic polymers account for 92% of all primary produced plastic ever made (1950–2015: 8,300 Mt). The largest groups are the polyolefins, with PE (36%), and PP (21%), PVC (12%), followed by PET, PUR, or PS (less than 10% each) [7, 9]. Biobased plastics (bioplastics) with increasing volumes emerged as non-fossil alternatives on the last decennium's plastic markets (Fig. 1A). Persistent biomass-derived plastic materials, non-biodegradable bioplastics derived from renewable resources represent 57% of all the bioplastics (2 Mt), including biobased PET, polyamides (PA), and PE [10]. Global bioplastics production capacities increase has been forecasted from around 2.1 Mt (biodegradable 1.3 Mt) in 2020 to approx. 2.9 Mt (1.8 Mt) in 2025, amounting to a 0.4% (2020) share of biodegradable plastics in general plastic waste streams [11].

Effective plastic recycling poses a significant challenge for sustainability, as a plastic polymer currently degrades each time it is recycled [12]. Technological solutions as part of a circular economy can form only part of more radical changes required in human behaviours like throw-away mentality symbolised by single-use consumer plastics or unnecessary packaging. Multilevel mitigation strategies to reduce the waste of natural resources started with policymakers banning and placing levies on single-use plastic consumer products to stimulate sustainable alternatives and changed consumer behaviour. Upcycling plastic waste from fossil sources in an open-loop process to biodegradable plastic and chemicals to valorise post-consumer plastic should be part of a rethinking towards a circular economy [13–15]. Essential for a circular economy is the intense utilisation of every side stream to minimise waste production or redundant CO_2_ release. Available recycling concepts are often not cost-competitive and produce polymers of lower quality. The biotechnological recycling supplemented with physicochemical techniques to tackle the more recalcitrant plastic polymers may promote new waste management strategies. Promising new value-chains for plastic waste and the increasing demand for recycled plastics by the multinational brand owners, driven mainly by the rising consumer awareness concerning sustainability issues, shall urge the private sector to invest in a circular economy. The partners of the MIX-UP consortium envision a better plastic future built on the '6 R' principles (rethink, refuse, reduce, reuse, recycle, replace) [16]. In the 2019 position paper "A circular economy for plastics", the European Commission explained its vision for a circular plastics system. The plastics should be produced using renewable energy and feedstocks. The plastic products should be designed to be used, reused, repaired and recycled (mechanically, chemically, biologically) so that the material streams in society are fully circular, keeping high value without posing risks for human health nor the environment [17].

### Chemical and mechanical plastic waste recycling

Crude oil and other fossil resources are the cost-effective lifeblood of the chemical industry and have been extensively used as energy and carbon feedstock for almost 90% of its products. One of the last boosts has been the shale gas-driven multi-billion investments into the U.S. chemical industry, leading to an acceleration of virgin plastics manufacturing [18]. For decades, the traditional economy of high-income countries followed the "take-make-dispose" strategy creating economic value by manufacturing and selling as many products as possible. The envisioned global transition to a circular economy initiated the founding of initiatives like, e.g. *The Global Plastics Alliance* or *Alliance to End Plastic Waste* [19, 20]. It activated private investors' investment or development banks into recycling technology to recover and create value from plastic waste.

The only widely applied large-scale technology to treat solid plastic waste is mechanical recycling. Limitations for mechanical recycling are temperature-sensitive plastics, composites, and thermosets that cannot be liquidised at high temperatures [21]. Differences in mechanical behaviour and thermal properties of all the different plastics require thorough sorting, washing to remove organic residues and shredding of the collected wastes. The melted and remoulded polymers are often blended with virgin plastics to correct for lost properties. Two of the most prominent commodity plastics, PET and PE, with annual EU market shares of about 8% and 30%, respectively, mainly used in packaging, are the only ones recovered by mechanical recycling [7, 21].

Chemical recycling has emerged as an alternative, promising technology to valorise plastic waste. Plastic wastes can be gasified into synthesis gas. Solvolytic processes may convert polymers into monomers and oligomers, subsequently re-polymerised after purification by, e.g. precipitation combined with filtration [22]. Pyrolysis (thermolysis) and hydrogenolysis using advanced catalysts can selectively produce gases, fuels, or waxes. The latter requires selective and efficient catalysts, preserving critical functional groups [21]. High-energy costs but low costs for competing virgin monomers from fossil-based feedstocks often make chemical recycling commercially unattractive [23]. Effective recycling processes within circular approaches should not only produce monomers for later polymerisation ("bottle to bottle") but rather focus on value-added products or intermediates for alternative supply chains. The upcycling of PE into long-chain alkyl aromatics ready to be sulfonated to make surfactants was reported [24]. Others described the synthesis of intermediary cyclic acetals, which are useful as solvents, fuel additives or monomers for polymers [25]. The greatest challenge is the chemical recycling of commingled plastic waste, as even small amounts of the various polymer contaminants may change the properties of the end-product. Therefore, chemical recycling requires often the use of pure waste feedstocks obtained only after resource-intensive sorting. The use of suitable compatibilisers for upcycling recovered polymer mixtures can overcome this problem [26–28].

Another feasible approach for PET/polylactate (PLA) polyester mixtures is using a molecular ruthenium catalyst for selective hydrogenolysis to separate the differing sorts of monomeric diols, and methanol, respectively, at varying reaction conditions (temperature, solvent) [29]. Alternatively, using pyrolysis oil in a naphtha cracker might close the carbon loop, but much of the beneficial molecular structure and plastic properties are lost in this option.

### Microbial and enzymatic plastics biotransformation

Facing the unabated growth of global plastic production and considering the shortcomings of traditional mechanical and chemical recycling technologies, biological depolymerisation and conversion technologies have been increasingly discussed, complementing end-of-life plastic treatment options. With a view to the economic circularity, selective removal of polymer-building blocks using enzymatic treatments under mild conditions and the ability to the selective recovery of monomers from mixed plastic substrates would be a real improvement [30–32]. Building blocks of plastic polymers can, in general, be divided into different major groups as (i) monomers with vinyl groups to produce PS, PE or PVC; (ii) bifunctional monomers with terminal hydroxyl, amine, or carboxyl groups to obtain polyesters or polyamides; (iii) diisocyanates for PUR [33]. In recent years, considerable progress concerning plastic polymers with hydrolysable groups in their backbones, as PET, PA, or PUR were reported, obtained mainly by polyaddition or polycondensation. Several studies described the ability of microorganisms and enzymes to degrade these plastics [34–47]. Typical enzymes are cutinases, lipases, and carboxylesterases [48]. The main challenge of enzymatic degradation is the fraction of plastic polymers based on persistent and robust chemical groups, which resist hydrolysis with common biological enzymes that are highly recalcitrant even under conditions favouring microbial processes. These polymers (e.g. PE, PP, PS, PVC) obtained by chain polymerisation comprise the major part of the plastic waste market and are generally considered non-biodegradable. The polymers possess extensive inert C–C backbone structures, are completely devoid of functional groups and might be only degraded by high-energy redox reactions [48]. Only a few enzymes have been reported to reduce the molar mass of PE and PS. Alkane hydroxylase AlkB, a hydroquinone peroxidase, laccases, and a laccase mediator system demonstrated C–C-bond cleavage via autooxidation mediated by putative radical mechanisms thought to occur randomly, generating a large diversity of short-chain scission products [4, 48–55]. In addition to the description of enzymatic activities towards PE and PS, several reports described their mineralisation to CO_2_ by insect larvae and their enteric microbiome. The latter potentially benefitting from the combined mechanical pretreatment and enzymatic hydrolysis [56–61]. Recently, biodegradation of PVC in the gut of *Tenebrio molitor* larvae has been described [62]. No biodegradation has been demonstrated so far for the highly recalcitrant polymer PP.

#### Mixed cultures in industrial applications

The application of microbial consortia in traditional foods, such as bread, soy sauce, cheese and wine, have been recorded for centuries. These bioprocesses were realised with naturally occurring mixed cultures. Mixed cultures were gradually replaced by pure cultures in fermentation processes to avoid contaminations by food spoilers or pathogenic microbes. Pure cultures have been the workhorses for biotechnological processes to produce bulk products like amino acids, antibiotics, enzymes or organic acids. Fermentations based on pure cultures usually require strict aseptic conditions, purified substrates, high operational energy costs, and gain in addition to the targeted product high concentrations of by-products in the form of biomass and potentially of organic acids or alcohols. The traditional strategy of consolidated bioprocessing integrates all bioconversion reactions in one step-bioprocesses using metabolically engineered whole-cell biocatalysts hosting all required functional genes in one consolidated strain.

Compared with the competing fossil-based chemical production, industrial biotechnology lacks cheap, readily available feedstocks to produce bulk biobased chemicals using highly specialised whole-cell biocatalysts as pure cultures. The main drawback for using lignocellulose, molasses, sludge or organic wastes as feedstock in pure-culture fermentations is the heterogeneity of the feedstocks, non-aseptic conditions and the high costs for substrate pre-treatments. Although mixed cultures as industrial microbiomes are well established in the fields of biofuels (biogas, bio-hydrogen, butanol-production), biobased chemicals, and biopolymers, the emphasis in industrial biotechnology still lies on pure cultures [63–66]. The specific advantages of mixed-culture fermentation compared with pure culture are (i) the possibility of utilising cheaper or mixed substrates (e.g. organic waste, lignocellulose, raw glycerol); (ii) the synergies of different enzymatic systems and combination of metabolic pathways of various microorganisms that can result in more efficient utilisation of substrates and a narrow production spectrum contributing to product purification; (iii) shorter development times for mixed-culture design compared with deep-genetic engineering to create universal "superbugs", and (iv) cost reduction, due to the high microbial diversity with non-sterile requirements [67]. An alternative for the latter is the use of robust extremophilic strains able to produce the target compounds (e.g. PHA) under simplified process conditions, in open unsterile, continuous fermentation facilities where most other organisms are unable to proliferate. The extremophiles based process seems to be suitable for simple growth on mixed degradation products, including fatty acids, plastic monomers and food wastes [68–71]. In mixed cultures and consortia exist in addition to intraspecies interactions, e.g. quorum sensing, interspecies interactions between cells of the different species. Metabolite effects like mutualism, synergy, and competition for nutrients in an ecological niche might affect metabolisms and the yield of fermentation target products [72–75].

#### *EU H2020 Project "From Plastic Waste to Plastic value using* Pseudomonas putida *Synthetic Biology" (P4SB): achieved results embedded in MIX-UP*

MIX-UP can, in part, build on the success of P4SB (grant no: 633962), an H2020 project in which several of the MIX-UP partners [RWTH-iAMB (coordinator, Aachen), University College Dublin, CIB-CSIC (Madrid), CNRS-University of Strasbourg; industrial partners: SOPREMA, Bioplastech] already worked together on plastic waste valorisation. The innovation radar has ranked P4SB as one of the top ten EU Biotechnology projects [76]. The main outcomes of P4SB regarding plastic hydrolysis are engineered PET degrading enzymes with significantly increased PET hydrolysing activity [77]. Furthermore, PUR hydrolases were identified [39]. In terms of monomer metabolism, *P. putida* strains for growth on all PET and PUR monomers tested could be isolated. However, efficient growth could not be achieved on all monomers. Subsequently, via genetic engineering, the P4SB partners could generate recombinant *P. putida* strains capable of efficient catabolism of ethylene glycol, terephthalic acid, and 1,4-butanediol [13, 14, 66, 78]. For the valorisation of plastic monomers, besides PHA synthesis, hydroxy alkanoyl oxy-alkanoic acids (HAA) synthesis has been successfully established. PHA synthesis could be shown on all PET and PUR plastic monomers [14]. Consolidated strains of *P. putida* engineered within P4SB for PET and PUR monomer metabolism will be used to benchmark the performance of defined mixed cultures in MIX-UP. Therefore, the strains will be combined with, e.g. engineered pseudomonads capable of metabolising oligo- and monomers from PS, PP, and PE degradation, and producing alternative novel biopolymers. In contrast to P4SB, MIX-UP focuses on consolidated bioprocesses in a combination of highly efficient enzymatic (pre-)treatment, defined mixed-culture biodegradation of released plastic monomers, additives and toxic constituents to biomass, value-added products, and building blocks using various engineered microbes. The increased interdisciplinary of the MIX-UP consortium was achieved by broadening the expertise’s in the following fields: intensive protein engineering (University Greifswald, RWTH-BIOTEC); plastic biodegradation (Nanjing Tech University, Beijing University of Chemical Technology); enzyme production upscaling (AB Enzymes, IPE); bacterial stress-response and directed evolution (Research Center Jülich); environmental education and innovative clean-up technology (everwave); extremophilic PHA-bioproduction (Tsinghua University); product separation and chemical catalysis (RWTH-AVT, ITMC).

## Project aim, concept, and approach

The core aim of MIX-UP project is to establish mixed plastic waste as standard second-generation feedstock for industrial biotechnology—plastic waste as a valuable resource. The bioconversion of unsorted, mixed plastic waste into value-added, sustainable biomaterials using heavily engineered enzyme mixtures for depolymerisation and mixed microbial cultures as whole-cell biocatalysts for biosynthesis is the way to achieve this goal as a contribution to the transition towards a low-fossil carbon circular bio-economy (Fig. 1B).

**Fig. 2:**
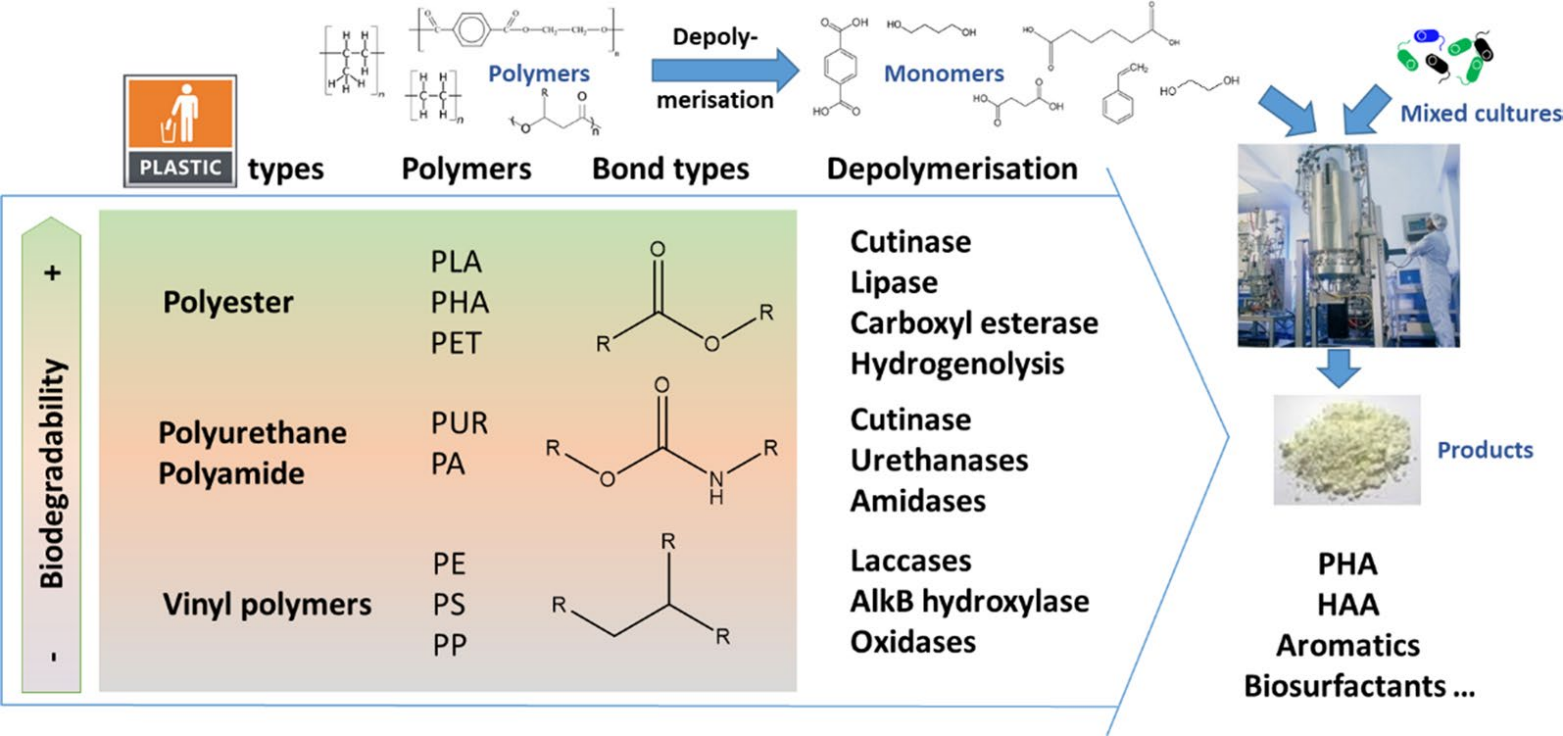
The generated MIX-UP workflow as combination of intensive protein engineering of different types of plastic depolymerising enzymes, metabolic engineering of defined mixed cultures and bioprocess-optimisation

The main idea of MIX-UP is to showcase a novel approach to the circularity of the plastic life cycle. The overall concept is depicted in Fig. 2. MIX-UP will develop and use engineered polymer hydrolyzing and oxidising enzymes to depolymerise the mechanically sheared mixed plastic waste (e.g*.* marine litter, household) into their monomeric components (biotic plastics depolymerisation). These enzymes will be expressed in mixed microbial cultures, synthesised in an optimised production reactor (enzyme production) or as envisioned in a subsequently consolidated bioprocess with simultaneously implemented whole-cell biocatalysts biodegradation. The released metabolites, additives (e.g. stabilisers, plasticisers, and colourants), plastic monomers, and oligomers from the various plastics types will be transferred to the bioreactor (mixed culture). Here the plastic derived feedstock is fed to dedicated microbial communities converting the substrate into central metabolites, which provide afterwards the building blocks for the synthesis of novel polymers (e.g. HAA, PHAs), products (biosurfactants) or building blocks for chemo-catalysis (Fig. 2). The approach follows the bow-tie structure of metabolism [79]. Finally, MIX-UP will tackle downstream processing and recovery of the product by, for example, conditional release of the intracellular products and separation. The recalcitrant process residues will be separated and subjected to chemical transformation, also cracking persistent ester bonds, synthesising valuable chemicals, and closing the cycle by subsequent re-entering of the bioprocesses. The entire bioprocess will be optimised, performing metabolic engineering in an integrated manner by considering the upstream (strain/microbiome development, protein engineering), midstream (fermentation), and downstream (recovery and purification) processes altogether.

MIX-UP targets the engineering of a new-to-nature biological route to convert mixed plastic waste to value-added bio-products, which will enable the recycling industry a qualitatively new dimension. Furthermore, when successful, mixed plastic wastes can be established as novel second-generation carbon sources for bio-products, aiding to solve the conflict of food *vs.* fuel that is pervasive in contemporary Industrial Biotechnology. Thus, through a combination of metabolic engineering of mixed cultures, intensive protein engineering and bioprocess-optimisation, MIX-UP will enable new value-chains within the framework of a sustainable knowledge-based bio-economy across sectors, including materials, chemicals, and environmental technologies. That will ultimately benefit the economy, environment, and society at large. The project has already produced a large number of publications that are available at the MIX-UP website www.mix-up.eu.

## Data Availability

Not applicable.

## Abbreviations

EU: European Union
MIX-UP: Mixed plastics biodegradation and upcycling using microbial communities
PP: Polypropylene
PE: Polyethylene
PUR: Polyurethane
PET: Polyethylene terephthalate
PS: Polystyrene
PA: Polyamide
PLA: Polylactate
PHA: Polyhydroxyalkanoate
PBS: Polybutylene succinate
TPS: Thermoplastic starch
PVC: Polyvinyl chloride
HAA: Hydroxylalkanoyloxy-alkanoic acids
Mt: Million metric tons
P4SB: From plastic waste to plastic value using *Pseudomonas putida* synthetic biology
H2020: Horizon 2020
*P. putida*: *Pseudomonas putida*

## References

[1] Ryan PG, Moore CJ, van Franeker JA, Moloney CL (2009). Monitoring the abundance of plastic debris in the marine environment. Phil Trans R Soc B.

[2] Dris R, Imhof H, Sanchez W, Gasperi J, Galgani F, Tassin B, Laforsch C (2015). Beyond the ocean: contamination of freshwater ecosystems with (micro-)plastic particles. Environ Chem.

[3] Abel SM, Primpke S, Int-Veen I, Brandt A, Gerdts G (2021). Systematic identification of microplastics in abyssal and hadal sediments of the Kuril Kamchatka trench. Environ Poll.

[4] Wright RJ, Erni-Cassola G, Zadjelovic V, Latva M, Christie-Oleza JA (2020). Marine plastic debris: a new surface for microbial colonization. Environ Sci Technol.

[5] Kane IA, Clare MA, Miramontes E, Wogelius R, Rothwell JJ, Garreau P, Pohl F (2020). Seafloor microplastic hotspots controlled by deep-sea circulation. Science.

[6] Brahney J, Hallerud M, Heim E, Hahnenberger M, Sukumaran S (2020). Plastic rain in protected areas of the United States. Science.

[7] PlasticsEurope. (2020). Plastics—the facts 2020.

[8] Law KL, Starr N, Siegler TR, Jambeck JR, Mallos NJ, Leonard GH (2020). The United States' contribution of plastic waste to land and ocean. Sci Adv.

[9] Geyer R, Jambeck JR, Law KL (2017). Production, use, and fate of all plastics ever made. Sci Adv.

[10] Andreessen C, Steinbüchel A (2018). Recent developments in non-biodegradable biopolymers: precursors, production processes, and future perspectives. Appl Microbiol Biotechnol.

[11] European-Bioplastics (2020) https://www.european-bioplastics.org/wp-content/uploads/2020/11/Global_Production_Capacity_Total_2019-2025.jpg. Accessed 29 July 2021.

[12] Stafford R, Jones PJS (2019). Viewpoint—ocean plastic pollution: a convenient but distracting truth?. Mar Pol.

[13] Blank LM, Narancic T, Mampel J, Tiso T, O'Connor K (2020). Biotechnological upcycling of plastic waste and other non-conventional feedstocks in a circular economy. Curr Opin Biotechnol.

[14] Tiso T, Narancic T, Wei R, Pollet E, Beagan N, Schröder K, Honak A, Jiang M, Kenny ST, Wierckx N, Perrin R, Avérous L, Zimmermann W, O'Connor K, Blank LM (2021). Towards bio-upcycling of polyethylene terephthalate. Metab Eng.

[15] Welsing G, Wolter B, Hintzen HMT, Tiso T, Blank LM (2021) Chapter Eighteen—Upcycling of hydrolyzed PET by microbial conversion to a fatty acid derivative. In: Weber G, Bornscheuer UT, Wei R (eds) Meth Enzymol, vol 648. Academic Press, Cambridge (MA), pp 391–421. 10.1016/bs.mie.2020.12.025 10.1016/bs.mie.2020.12.025 33579413

[16] Wei R, Tiso T, Bertling J, O'Connor K, Blank LM, Bornscheuer UT (2020). Possibilities and limitations of biotechnological plastic degradation and recycling. Nat Catal.

[17] Crippa M, De Wilde B, Koopmans R, Leyssens J, Muncke J, Ritschkoff A-C, van Doorsselaer K, Velis C, Wagner M (2019). A circular economy for plastics—insights from research and innovation to inform policy and funding decisions. European Union.

[18] Borrelle SB, Ringma J, Law KL, Monnahan CC, Lebreton L, McGivern A, Murphy E, Jambeck J, Leonard GH, Hilleary MA, Eriksen M, Possingham HP, De Frond H, Gerber LR, Polidoro B, Tahir A, Bernard M, Mallos N, Barnes M, Rochman CM (2020). Predicted growth in plastic waste exceeds efforts to mitigate plastic pollution. Science.

[19] Alliance to End Plastic Waste (2021) https://www.endplasticwaste.org. Accessed 28 Apr 2021.

[20] Global Plastics Alliance, Marine Litter Solutions (2021) https://www.marinelittersolutions.com. Accessed 28 Apr 2021.

[21] Garcia JM, Robertson ML (2017). The future of plastics recycling. Science.

[22] Walker TW, Frelka N, Shen Z, Chew AK, Banick J, Grey S, Kim MS, Dumesic JA, Van Lehn RC, Huber GW (2020). Recycling of multilayer plastic packaging materials by solvent-targeted recovery and precipitation. Sci Adv.

[23] Weckhuysen BM (2020). Creating value from plastic waste. Science.

[24] Zhang F, Zeng M, Yappert RD, Sun J, Lee Y-H, LaPointe AM, Peters B, Abu-Omar MM, Scott SS (2020). Polyethylene upcycling to long-chain alkylaromatics by tandem hydrogenolysis / aromatization. Science.

[25] Beydoun K, Klankermayer J (2020). Efficient plastic waste recycling to value-added products by integrated biomass processing. Chemsuschem.

[26] Hamad K, Kaseem M, Deri F (2013). Recycling of waste from polymer materials: an overview of the recent works. Pol Deg Stab.

[27] Eagan JM, Xu J, Di Girolamo R, Thurber CM, Macosko CW, LaPointe AM, Bates F, Coates GW (2017). Combining polyethylene and polypropylene: enhanced performance with PE/iPP multiblock polymers. Science.

[28] Creton C (2017). Molecular stitches for enhanced recycling of packaging. Science.

[29] Westhues S, Idel J, Klankermayer J (2018). Molecular catalyst systems as key enablers for tailored polyesters and polycarbonate recycling concepts. Sci Adv.

[30] Wei R, Zimmermann W (2017). Microbial enzymes for the recycling of recalcitrant petroleum-based plastics: how far are we?. Microb Biotechnol.

[31] Wierckx N, Narancic T, Eberlein C, Wei R, Drzyzga O, Magnin A, Ballerstedt H, Kenny ST, Pollet E, Avérous L, O'Connor KE, Zimmermann W, Heipieper HJ, Prieto A, Jiménez J, Blank LM (2018) Plastic biodegradation: Challenges and opportunities. In: Steffan R (ed) Consequences of microbial interactions with hydrocarbons, oils, and lipids: Biodegradation and bioremediation. Springer, Cham. 10.1007/978-3-319-44535-9_23-1

[32] Wierckx N, Prieto AM, Pomposiello P, de Lorenzo V, O'Connor K, Blank LM (2015). Plastic waste as a novel substrate for industrial biotechnology. Microb Biotechnol.

[33] Schaffer S, Haas T (2014). Biocatalytic and fermentative production of α, ω-bifunctional polymer precursors. Org Proc Res Dev.

[34] Cregut M, Bedas M, Durand MJ, Thouand G (2013). New insights into polyurethane biodegradation and realistic prospects for the development of a sustainable waste recycling process. Biotechnol Adv.

[35] Schmidt J, Wei R, Oeser T, Dedavid e Silva L, Breite D, Schulze A, Zimmermann W (2017). Degradation of polyester polyurethane by bacterial polyester hydrolases. Polymers.

[36] Wei R, Zimmermann W (2017). Biocatalysis as a green route for recycling the recalcitrant plastic polyethylene terephthalate. Microb Biotechnol.

[37] Knott BC, Erickson E, Allen MD, Gado JE, Graham R, Kearns FL, Pardo I, Topuzlu E, Anderson JJ, Austin HP, Dominick G, Johnson CW, Rorrer NA, Szostkiewicz CJ, Copié V, Payne CM, Woodcock HL, Donohoe BS, Beckham GT, McGeehan JE (2020). Characterization and engineering of a two-enzyme system for plastics depolymerization. PNAS.

[38] Tournier V, Topham CM, Gilles A, David B, Folgoas C, Moya-Leclair E, Kamionka E, Desrousseaux ML, Texier H, Gavalda S, Cot M, Guémard E, Dalibey M, Nomme J, Cioci G, Barbe S, Chateau M, André I, Duquesne S, Marty A (2020). An engineered PET depolymerase to break down and recycle plastic bottles. Nature.

[39] Magnin A, Pollet E, Perrin R, Ullmann C, Persillon C, Phalip V, Avérous L (2019). Enzymatic recycling of thermoplastic polyurethanes: Synergistic effect of an esterase and an amidase and recovery of building blocks. Waste Manag.

[40] Deguchi T, Kitaoka Y, Kakezawa M, Nishida T (1998). Purification and characterization of a nylon-degrading enzyme. Appl Environ Microbiol.

[41] Yoshida S, Hiraga K, Takehana T, Taniguchi I, Yamaji H, Maeda Y, Toyohara K, Miyamoto K, Kimura Y, Oda K (2016). A bacterium that degrades and assimilates poly(ethylene terephthalate). Science.

[42] Oda M, Numoto N, Bekker G-J, Kamiya N, Kawai F (2021) Chapter Eight—Cutinases from thermophilic bacteria (actinomycetes): From identification to functional and structural characterization. In: Weber G, Bornscheuer UT, Wei R (eds) Meth Enzymol, vol 648. Academic Press, Cambridge (MA), pp 159–85. 10.1016/bs.mie.2020.12.031 10.1016/bs.mie.2020.12.031 33579402

[43] Magnin A, Pollet E, Avérous L (2021) Chapter Fifteen—Characterization of the enzymatic degradation of polyurethanes. In: Weber G, Bornscheuer UT, Wei R (eds) Meth Enzymol, vol 648. Academic Press, Cambridge (MA), pp 317–336. 10.1016/bs.mie.2020.12.011 10.1016/bs.mie.2020.12.011 33579410

[44] Yan F, Wei R, Cui Q, Bornscheuer UT, Liu Y-J (2020). Thermophilic whole-cell degradation of polyethylene terephthalate using engineered *Clostridium thermocellum*. Microb Biotechnol.

[45] Meyer-Cifuentes IE, Werner J, Jehmlich N, Will SE, Neumann-Schaal M, Öztürk B (2020). Synergistic biodegradation of aromatic-aliphatic copolyester plastic by a marine microbial consortium. Nat Commun.

[46] Magnin A, Pollet E, Phalip V, Avérous L (2020). Evaluation of biological degradation of polyurethanes. Biotechnol Adv.

[47] Li Z, Wei R, Gao M, Ren Y, Yu B, Nie K, Xu H, Liu L (2020). Biodegradation of low-density polyethylene by *Microbulbifer hydrolyticus* IRE-31. J Environ Manag.

[48] Inderthal H, Tai SL, Harrison STL (2020). Non-hydrolyzable plastics—an interdisciplinary look at plastic bio-oxidation. Trends Biotechnol.

[49] Krueger MC, Harms H, Schlosser D (2015). Prospects for microbiological solutions to environmental pollution with plastics. Appl Microbiol Biotechnol.

[50] Krueger MC, Seiwert B, Prager A, Zhang S, Abel B, Harms H, Schlosser D (2017). Degradation of polystyrene and selected analogues by biological Fenton chemistry approaches: Opportunities and limitations. Chemosphere.

[51] Yoon MG, Jeon HJ, Kim NM (2012). Biodegradation of polyethylene by a soil bacterium and AlkB cloned recombinant cell. J Bioremed Biodegrad.

[52] Nakamiya K, Sakasita G, Ooi T, Kinoshita S (1997). Enzymatic degradation of polystyrene by hydroquinone peroxidase of *Azotobacter beijerinckii* HM121. J Ferm Bioeng.

[53] Santo M, Weitsman R, Sivan A (2013). The role of the copper-binding enzyme—laccase—in the biodegradation of polyethylene by the actinomycete *Rhodococcus ruber*. Int Biodeter Biodegr.

[54] Kowalczyk A, Chyc M, Ryszka P, Latowski D (2016). *Achromobacter xylosoxidans* as a new microorganism strain colonizing high-density polyethylene as a key step to its biodegradation. Environ Sci Poll Res.

[55] Erni-Cassola G, Wright RJ, Gibson MI, Christie-Oleza JA (2020). Early Colonization of weathered polyethylene by distinct bacteria in marine coastal seawater. Microb Ecol.

[56] Yang Y, Yang J, Wu WM, Zhao J, Song Y, Gao L, Yang R, Jiang L (2015). Biodegradation and mineralization of polystyrene by plastic-eating mealworms: Part 1. Chemical and physical characterization and isotopic tests. Environ Sci Technol.

[57] Yang Y, Yang J, Wu WM, Zhao J, Song Y, Gao L, Yang R, Jiang L (2015). Biodegradation and mineralization of polystyrene by plastic-eating mealworms: Part 2. Role of gut microorganisms. Environ Sci Technol.

[58] Yang S-S, Brandon AM, Andrew Flanagan JC, Yang J, Ning D, Cai S-Y, Fan H-Q, Wang Z-Y, Ren J, Benbow E, Ren N-Q, Waymouth RM, Zhou J, Criddle CS, Wu W-M (2018). Biodegradation of polystyrene wastes in yellow mealworms (larvae of *Tenebrio molitor* Linnaeus): factors affecting biodegradation rates and the ability of polystyrene-fed larvae to complete their life cycle. Chemosphere.

[59] Kim HR, Lee HM, Yu HC, Jeon E, Jeon S, Li J, Kim D-H (2020). Biodegradation of polystyrene by *Pseudomonas* sp. isolated from the gut of superworms (larvae of *Zophobas atratus*). Environ Sci Technol.

[60] Peng B-Y, Su Y, Chen Z, Chen J, Zhou X, Benbow ME, Criddle CS, Wu W-M, Zhang Y (2019). Biodegradation of polystyrene by dark (*Tenebrio obscurus*) and yellow (*Tenebrio molitor*) mealworms (*Coleoptera: Tenebrionidae*). Environ Sci Technol.

[61] Wu W-M, Criddle CS (2021) Chapter Five—Characterization of biodegradation of plastics in insect larvae. In: Weber G, Bornscheuer UT, Wei R (eds) Meth Enzymol, vol 648. Academic Press, Cambridge (MA), pp 95–120. 10.1016/bs.mie.2020.12.029 10.1016/bs.mie.2020.12.029 33579419

[62] Peng B-Y, Chen Z, Chen J, Yu H, Zhou X, Criddle CS, Wu W-M, Zhang Y (2020). Biodegradation of polyvinyl chloride (PVC) in *Tenebrio molitor* (*Coleoptera: Tenebrionidae*) larvae. Environ Internat.

[63] Dias JM, Lemos PC, Serafim LS, Oliveira C, Eiroa M, Albuquerque MG, Ramos AM, Oliveira R, Reis MA (2006). Recent advances in polyhydroxyalkanoate production by mixed aerobic cultures: from the substrate to the final product. Macromol Biosci.

[64] Dietz D, Zeng AP (2014). Efficient production of 1,3-propanediol from fermentation of crude glycerol with mixed cultures in a simple medium. Bioproc Biosyst Eng.

[65] Moita R, Freches A, Lemos PC (2014). Crude glycerol as feedstock for polyhydroxyalkanoates production by mixed microbial cultures. Water Res.

[66] Utomo RNC, Li W-J, Tiso T, Eberlein C, Doeker M, Heipieper HJ, Jupke A, Wierckx N, Blank LM (2020). Defined microbial mixed culture for utilization of polyurethane monomers. ACS Sust Chem Eng.

[67] Jiang L-L, Zhou J-J, Quan C-S, Xiu Z-L (2017). Advances in industrial microbiome based on microbial consortium for biorefinery. Biores Bioproc.

[68] Zheng Y, Chen JC, Ma YM, Chen GQ (2020). Engineering biosynthesis of polyhydroxyalkanoates (PHA) for diversity and cost reduction. Metab Eng.

[69] Chen GQ, Jiang XR (2018). Next generation industrial biotechnology based on extremophilic bacteria. Curr Opin Biotechnol.

[70] Tan D, Wang Y, Tong Y, Chen G-Q (2021). Grand challenges for industrializing polyhydroxyalkanoates (PHAs). Trends Biotechnol.

[71] Jiang XR, Yan X, Yu LP, Liu XY, Chen GQ (2021). Hyperproduction of 3-hydroxypropionate by *Halomonas bluephagenesis*. Nat Commun.

[72] Federle MJ, Bassler BL (2003). Interspecies communication in bacteria. J Clin Invest.

[73] Nowak MA (2006). Five rules for the evolution of cooperation. Science.

[74] Du R, Yan J, Li S, Zhang L, Zhang S, Li J, Zhao G, Qi P (2015). Cellulosic ethanol production by natural bacterial consortia is enhanced by *Pseudoxanthomonas taiwanensis*. Biotechnol Biofuels.

[75] Stolyar S, Van Dien S, Hillesland KL, Pinel N, Lie TJ, Leigh JA, Stahl DA (2007). Metabolic modeling of a mutualistic microbial community. Mol Syst Biol.

[76] DECHEMA Gesellschaft für Chemische Technik und Biotechnologie e.V. (2019) KETBIO. Top Ten EU Biotech projects. https://ketbio.eu/KETBIO_Parade. Accessed 23 Nov 2020

[77] Wei R, Breite D, Song C, Gräsing D, Ploss T, Hille P, Schwerdtfeger R, Matysik J, Schulze A, Zimmermann W (2019). Biocatalytic degradation efficiency of postconsumer polyethylene terephthalate packaging determined by their polymer microstructures. Adv Sci.

[78] Li W-J, Narancic T, Kenny ST, Niehoff P-J, O'Connor K, Blank LM, Wierckx N (2020). Unraveling 1,4-butanediol metabolism in *Pseudomonas putida* KT2440. Front Microbiol.

[79] Sudarsan S, Dethlefsen S, Blank LM, Siemann-Herzberg M, Schmid A (2014). The functional structure of central carbon metabolism in *Pseudomonas putida* KT2440. Appl Environ Microbiol.

